# Comparison between CDC and WHO BMI z-score and their relation with metabolic risk markers in Northern Portuguese obese adolescents

**DOI:** 10.1186/s13098-015-0022-7

**Published:** 2015-04-08

**Authors:** Henrique Nascimento, Cristina Catarino, Denisa Mendonça, Pedro Oliveira, Ana Inês Alves, Ana Filipa Medeiros, Petronila Rocha Pereira, Carla Rêgo, Helena Ferreira Mansilha, Luísa Aires, Jorge Mota, Alexandre Quintanilha, Alice Santos-Silva, Luís Belo

**Affiliations:** Biological Science Department, Faculty of Pharmacy, University of Porto, Porto, Portugal; Instituto de Biologia Molecular e Celular (Institute for Molecular and Cell Biology), Universidade do Porto, Porto, Portugal; Instituto de Investigação e Inovação em Saúde (Institute for Research and Innovation in Health), Universidade do Porto, Porto, Portugal; Population Study Department, Biomedical Sciences Institute Abel Salazar (ICBAS), University of Porto, Porto, Portugal; Research Centre in Physical Activity, Health and Leisure (CIAFEL), Faculty of Sports, University of Porto, Porto, Portugal; Health Science Research Centre, University of Beira Interior, Covilhã, Portugal; Children and Adolescent Centre, CUF Hospital; Center for Health Technology and Services Research (CINTESIS), Faculty of Medicine, University of Porto, Porto, Portugal; Childhood and Adolescence Department/Paediatric Service of Porto Hospital Centre, Porto, Portugal; University Institute of Maia (ISMAI), S. Pedro Avioso, Portugal; Biomedical Sciences Institute Abel Salazar (ICBAS), University of Porto, Porto, Portugal

**Keywords:** BMI z-score, CDC, WHO, Metabolic syndrome, Insulin resistance

## Abstract

**Background:**

Growth-curves are an important tool for evaluating the anthropometric development in pediatrics. The different growth-curves available are based in different populations, what leads to different cut-offs.

Pediatric obesity tracks into adulthood and is associated with increased cardiovascular risk. The accurate assessment of a child nutritional status using growth-curves can indicate individuals that are either obese or in risk of becoming obese, allowing an early intervention. Moreover, the association between the data obtained from growth-curves with specific metabolic risk factors further highlights the importance of these charts.

This study aimed to evaluate the associations between body mass index z-score (BMIzsc), determined using the growth-curves from the Centre for Disease Control and Prevention (CDC) and from the World Health Organization (WHO), with cardiovascular risk factors, represented here by metabolic syndrome (MS) and insulin resistance (IR) related parameters.

The study involved 246 obese adolescents (10–18 years, 122 females). MS was defined according to the International Diabetes Federation. IR was considered for HOMA-IR greater than 2.5.

**Findings:**

No difference between both BMIzsc in identifying MS was noticeable by a ROC analysis. For both indexes the area-under-the-curve increased for older groups, particularly for males.

CDC-BMIzsc was the best predictor of MS by logistic regression when all population was considered, however MS was better predicted by WHO-BMIzsc for females and by CDC-BMIzsc for males. Younger girls and older boys were in increased risk for MS. Similar results were obtained for IR.

**Conclusions:**

A significant difference between the two BMIzsc regarding their association with MS and IR was not clear, being these associations weaker in younger individuals.

## Findings

### Introduction

Pediatric obesity is an important problem in Portugal with almost one third of Portuguese children and adolescents being overweight or obese [1-3]. As obesity is associated with increased metabolic and cardiovascular risk [4], it is important to accurately evaluate the nutritional status in pediatrics and identify individuals in risk in order to intervene as soon as possible.

During childhood body mass index (BMI) is adjusted for age and gender defining the BMI z-score (BMIzsc). Two of the most used BMI-based criteria to evaluate the nutritional status are those from the Centre for Disease Control and Prevention (CDC) and from the World Health Organization (WHO) [5]. These criteria have different base population, leading to different growth-curves and cut-offs. In fact, for a given population, WHO-BMIzsc associates with increased percentage of obese and reduced percentage of under-weight when compared to CDC-BMIzsc [1].

In Portugal the CDC criterion was recommend by the Portuguese Ministry of Health until 2012, being the criterion from WHO the recommended since 2013 [6]. It is necessary to evaluate how this change influences this tool efficacy in characterizing the nutritional status.

Furthermore, rises in both BMIzsc, have been associated with increased cardiovascular risk factors [7,8]. Nevertheless, there is limited information regarding how the relation between BMIzsc and biochemical variables is affected by this change, and if there is any statistical advantage in choosing one criterion over the other.

This study aimed to evaluate the differences in BMIzsc determined using the criteria of CDC and WHO [9,10], and their relation with cardiovascular risk factors, represented here by metabolic syndrome (MS) and insulin resistance (IR) related parameters.

## Methods

The study involved 246 obese adolescents (10–18 years, 122 females) identified from medical records from two Porto hospitals (Portugal). Anthropometric and biochemical markers were determined as described elsewhere [11]. Participant’s BMIzsc were classified according to WHO [10] and CDC [9] criteria.

The participants were classified as presenting or not MS according to the International Diabetes Federation [12].

The population was also divided in presenting or not IR according to having a homeostasis model of insulin resistance (HOMA-IR) greater than 2.5 [13,14].

The distributions of continuous variables were analyzed using Kolmogorov-Smirnov tests to assess significant departures from normality. Normally distributed variables are presented as mean ± SD (standard deviation) and those non-normally distributed are presented as median (interquartile range). Variables non-normally distributed were log transformed before further analysis.

Comparisons between two groups were performed using Student’s unpaired t-test. The association between categorical variables was analyzed using chi-squared test and Fisher’s exact test.

The strength of the association between the variables was estimated by Pearson correlation coefficient.

To evaluate the agreement between the BMIzsc defined according to the WHO and the CDC criteria a Bland–Altman analysis was performed.

The population was divided according to the following age groups: Group 1: males < 13 and females < 12 years; Group 2: males ≥ 13 and females ≥ 12 years.

To evaluate the association of MS with the two BMIzsc criteria a receiver operating characteristic (ROC) analysis was performed.

A stepwise logistic regression analysis with MS or IR as the dependent variable, and an entry criteria of *P* <0.05, was also performed involving the two BMIzsc as independent variables, while controlling for age and gender.

Both the ROC and the logistic regression analysis were repeated while dividing the population according to gender and age groups.

Statistical analysis was performed using the Statistical Package for Social Sciences (SPSS, version 21.0) for Windows (SPSS Inc., Armonk, NY, USA). Statistical significance was accepted at *P* less than 0.05.

## Results

The population characteristics are resumed in Table 1. The individual correlations between the two BMIzsc and the different metabolic markers were similar being the highest correlations found with waist-to-height ratio and insulin. Regarding the MS groups, there was no difference in gender and Tanner stage distributions.

**Table 1:** **Comparison between clinical and biochemical data of obese children and adolescents based on the presence of metabolic syndrome and the correlation of anthropometric and metabolic variables with BMI z-score defined according to CDC or WHO criteria**

				**Correlation with BMI z-score** ^**1)**^	
	**Without MS**	**With MS**	***P*** ^**2)**^	**WHO**	**CDC**
Number of participants (%)	185 (75.2)	61 (24.8)	-	-	-
Females, n (%)	97 (52.4)	25 (41.0)	0.141	-	-
Pubertal Stage (pre-pub (%))^3)^	30 (16.2)	8 (13.1)	0.684	-	-
Age (years)	12.7 ± 1.8	13.4 ± 2.1	0.017	0.057	0.122
Height (cm)	158.5 ± 8.9	162.8 ± 9.9	0.002	0.189**	0.277**
Weight (kg)	78.0 (68.0-89.0)	90.2 (76.0-113.5)	<0.001	0.673**	0.726**
BMI (kg/m^2^)	30.55 (28.15-34.08)	34.52 (30.20-39.14)	<0.001	0.848**	0.865**
BMI z-score (CDC)	2.20 ± 0.29	2.43 ± 0.31	<0.001	0.933**	-
BMI z-score (WHO)	3.00 ± 0.62	3.51 ± 0.86	<0.001	-	0.933**
Waist circumference (cm)	99.0 (91.8-106.5)	105.0 (96.5-118.0)	<0.001	0.736**	0.778**
Waist / Height	0.630 ± 0.066	0.668 ± 0.081	0.002	0.794**	0.783**
**Blood pressure**					
Systolic (mmHg)^4)^	116 ± 12	135 ± 13	<0.001	0.323**	0.360**
Diastolic (mmHg)^4)^	63 ± 8	70 ± 12	0.001	0.267**	0.218**
**Lipid profile**					
TG (mg/dl)	71.0 (53.1-100.6)	126.7 (81.5-181.5)	<0.001	0.186**	0.185**
HDLc (mg/dl)	43.0 (38.3-49.0)	35.0 (32.0-38.4)	<0.001	−0.215**	−0.223**
TC (mg/dl)	150.8 (134.5-174.0)	164.0 (142.0-189.2)	0.038	0.105	0.084
LDLc (mg/dl)	92.0 (76.0-109.8)	101.0 (83.5-119.4)	0.055	0.145*	0.126*
**Glucose metabolism**					
Glucose (mg/dl)	83.0 (77.0-89.6)	88.0 (80.0-93.8)	0.005	0.043	0.046
Insulin (μU/ml)	15.9 (12.2-22.1)	23.3 (14.6-33.3)	<0.001	0.421**	0.440**
HOMA-IR	3.18 (2.43-4.52)	4.94 (3.34-7.06)	<0.001	0.410**	0.429**
**Inflammatory mediator**					
CRP (mg/l)^5)^	1.61 (0.84-4.05)	2.30 (1.00-4.59)	0.072	0.326**	0.302**

*, P < 0.05; **, P < 0.001; ^1)^Pearson correlations; ^2)^With MS *vs.* Without MS groups; ^3)^Pre-pubertal defined as Tanner stage = 1; ^4)^With MS n = 45, without MS n = 172; ^5)^With MS n = 60, without MS, n = 180; BMI, body mass index; CDC, Center for Disease Control and Prevention; CRP, c-reactive protein; HDLc, high density lipoprotein cholesterol; HOMA-IR, homeostasis model of assessment-insulin resistance; LDLc, low density lipoprotein cholesterol; MS, metabolic syndrome; pre-pub, pre-pubertal; TC, total cholesterol; TG, triglycerides; WHO, World Health Organization.

**Figure 1:**
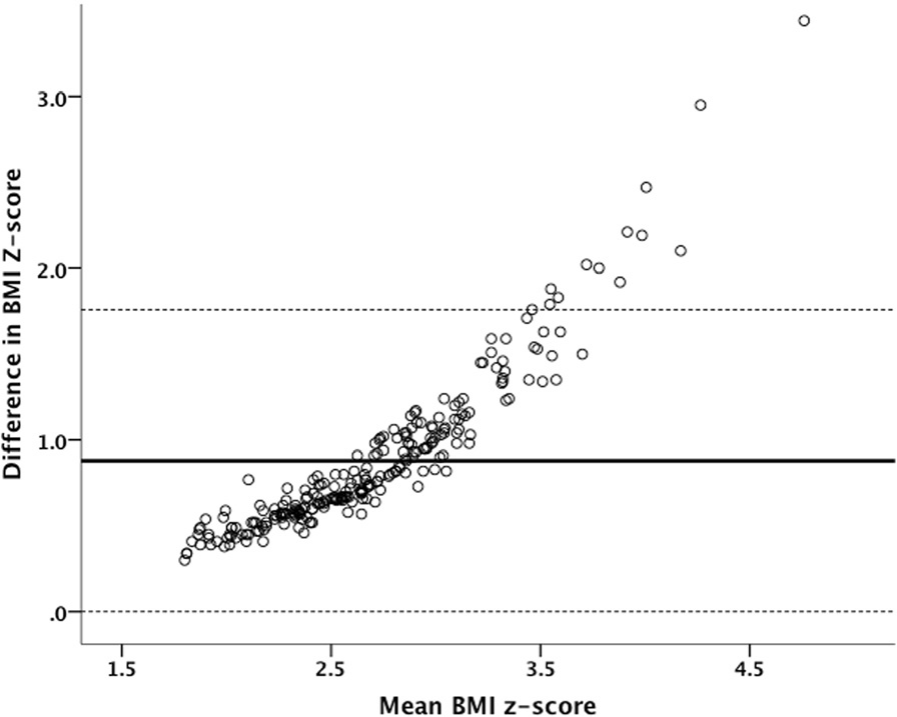
**Bland–Altman plot of the difference in BMI z-score measured according to the World Health Organization (WHO) and the Center for Disease Control and Prevention (CDC) against the average of the measured BMI z-score.** Horizontal lines represent mean ± 2 standard deviation. Difference was calculated WHO BMI z-score – CDC BMI z-score.

Figure 1 presents a Bland–Altman plot between WHO and CDC BMIzsc. This type of analysis can be used to evaluate the agreement between two methods/assays that quantify the same variable. It can be observed that the difference between the scores increases with the increase of their average; thus, these scores will classify subjects differently, especially for larger BMIzsc.

No difference between both BMIzsc in identifying MS was noticeable concerning ROC analysis (Table 2). There was also no difference when the same analysis was repeated further stratifying the population according to gender and age groups. BMIzsc from both criteria did not seem to associate with MS for the younger individuals, both when considered together or separated by gender (particularly in males), contrarily to older individuals (Table 2).

**Table 2:** **Association of BMI z-score calculated according to WHO and CDC criteria with metabolic syndrome and insulin resistance (based on receiver operating characteristic (ROC) and logistic regression analysis)**

	**ROC**		**Logistic Regression** ^**1)**^
	**WHO**	**CDC**	
	**AUC**		**expB (95% CI)**
**Metabolic syndrome**			
Total population (n = 246; MS = 61)	0.680**	0.703**	CDC 15.66** (5.16-47.59)
Male Age group 1 (n = 57; MS = 9)	0.508	0.543	CDC 17.55** (3.90-79.06); Age* 3.65 (1.14-11.70)
Male Age group 2 (n = 67; MS = 27)	0.763**	0.788**	
Female Age group 1 (n = 39; MS = 12)	0.642	0.636	WHO 2.76* (1.36-5.60); Age* 0.39 (0.15-0.99)
Female Age group 2 (n = 83; MS = 13)	0.693*	0.664	
**Insulin resistance**			
Total population (n = 246; IR = 190)	0.655**	0.680**	CDC 10.86** (3.64-32.46)
Male Age group 1 (n = 57; IR = 39)	0.551	0.582	CDC 8.18* (1.97-33.96)
Male Age group 2 (n = 67; IR = 53)	0.720*	0.739*	
Female Age group 1 (n = 39; IR = 32)	0.777**	0.777**	CDC 21.77** (3.76-126.02)
Female Age group 2 (n = 83; IR = 66)	0.696*	0.689*	

*, *P* < 0.05; **, *P* < 0.001; ^1)^adjusted for age groups; insulin resistance defined as HOMA-IR ≥ 2.5; metabolic syndrome defined according to the International Diabetes Federation; age group 1: males <13 years and females <12 years; age group 2: males ≥ 13 years, females ≥ 12 years; AUC, area under the curve; CDC, Center for Disease Control and Prevention; CI, confidence interval; IR, insulin resistance; ROC, Receiver operating characteristic; MS, metabolic syndrome; WHO, World Health Organization.

The variable that entered the model in the stepwise logistic regression analysis for MS with the two BMIzsc as independent variables when all population was considered, while controlling for age and gender, was CDC-BMIzsc (Table 2). Afterwards the analysis was repeated according to gender, being the presence of MS better predicted by WHO-BMIzsc for females, while by CDC-BMIzsc for males. When the analysis was made considering the age groups, no variable entered the model for the younger subjects, both when considered all together or separated by genders. For the older group the associations mimicked the ones seen for the whole population (data not shown).

The logistic regression analysis also revealed that younger girls are in a slightly increased risk for MS, while for males the effect of aging is the opposite, with older boys presenting a worsening of the odds ratio. Puberty interfere with the metabolic status differently according to gender. Indeed, features of MS, as lipid profile, fat distribution, blood pressure, insulin sensitivity, vary through pediatric ages, with a particularly strong fluctuation during puberty, while pre-pubertal sexual differences are usually moderate or absent [12,15,16]. After puberty, the differences in sexual hormones seem to relate to the less atherogenic lipid profile and improved inflammatory status presented by females when compared to males. These changes in inflammatory factors, particularly mediators closely associated with IR and MS could explain part of the gender-related differences. In fact, post-puberty girls (*vs.* boys) present an inflammatory status, characterized by increased adiponectin and reduced C-reactive protein, that is linked with reduced IR and improved plasmatic lipids [17,18].

A similar analysis was made replacing MS by IR and dividing the population in presenting or not IR according to having a HOMA-IR greater than 2.5 [13,14]. The results were similar to the ones obtained for MS, nevertheless, CDC-BMIzsc was the best predictor of IR for all groups (except for young males, for whom neither BMIzsc entered the model, data not shown) (Table 2).

### Limitations of the study

This study presents some limitations, namely: it was limited to a geographical region (Northern Portugal) and only obese individuals were included. In this way, it is not clear if the present results will apply to different populations or normal weight individuals.

## Conclusion

Concluding, it does not seem to exist a significant difference between the BMIzsc calculated according to the WHO or the CDC criteria regarding their relation with metabolic markers or power to predict MS or IR in obese Portuguese adolescents. In the younger individuals, the association between BMIzsc and MS or IR seems to be weaker or not yet present, especially in males. For older individuals there might be a slight better association between CDC-BMIzsc and IR for all groups, while MS seems to associate better with WHO-BMIzsc for females and with CDC-BMIzsc for males. An increased risk for MS for older males and younger females also appears to be present, highlighting the importance of an early intervention. This is, to the best of our knowledge, the first paper to describe the increased risk of MS for younger female adolescents. A protective effect of female sexual hormones, particularly estrogens, against cardiovascular risk has been long discussed [19]. In fact, it seems to exist a down regulation of visceral adipose tissue related oxidative stress and inflammation by estrogens, particularly in obese individuals [19,20]. Nevertheless, other articles point to a negative effect of puberty on cardiovascular risk in both males and females [21]. Further studies are needed to clarify the influence of puberty itself and pre-pubertal development in future cardiovascular disease risk.

The growth-curves are an everyday instrument for the pediatricians to follow the child/adolescent development. Although the WHO criterion was built to be better adjusted to populations with different backgrounds, it does not seem to present many differences to CDCs’ regarding the relation with metabolic risk markers. Moreover, the use of different criteria can lead to different epidemiologic data even without effective variation in the population being present. Thus, studies before and after the adoption of the new criteria should be carefully compared.

## Abbreviations

BMI: Body mass index
BMIzsc: BMI z-score
CDC: Centre for Disease Control and Prevention
HOMA-IR: Homeostasis model assessment of insulin resistance
IR: Insulin resistance
ROC: Receiver operating characteristic
MS: Metabolic syndrome
WHO: World Health Organization
